# Subthreshold micropulse laser therapy for early postoperative macular thickening following surgical removal of epiretinal membrane

**DOI:** 10.1186/s12886-024-03365-1

**Published:** 2024-03-05

**Authors:** Hongjie Lin, Zijing Huang, Dingguo Huang, Dezhi Zheng, Peimin Lin, Yangxuan Lin, Weiqi Chen

**Affiliations:** Joint Shantou International Eye Center of Shantou University and The Chinese University of Hong Kong, 69 North Dongxia Rd, Shantou, Guangdong 515041 China

**Keywords:** Subthreshold micropulse laser, Epiretinal membrane, Macular edema, Membrane peeling, Central subfield thickness

## Abstract

**Background:**

This study aimed to investigate the functional and anatomical outcomes of subthreshold micropulse laser (SMPL) therapy in eyes with early postoperative macular thickening after idiopathic epiretinal membrane (iERM) removal.

**Methods:**

This was a prospective and interventional study. Forty-eight eyes from 48 patients with macular edema at 1 month after iERM removal were randomly divided into two groups. Patients in the SMPL group (*n* = 24) received SMPL therapy while no special intervention was used for the observation group (*n* = 24). Baseline demographic data and clinical findings before and at 1 and 3 months after SMPL treatment or observation, including best-corrected visual acuity (BCVA) and the changes in central subfield thickness (CST) and average macular thickness (AMT), were analyzed.

**Results:**

An improvement in BCVA with a decrease in CST and AMT from baseline to the 3-month follow-ups were observed in both SMPL and observation groups. No significant difference in BCVA was observed between the SMPL group and observation group either in the 1-month (0.26 [0.15, 0.52] vs. 0.26 [0.15, 0.39], *P* = 0.852) or the 3-month (0.15 [0.10, 0.30] vs. 0.23 [0.15, 0.30], *P* = 0.329) follow-up. There was a greater reduction in CST in the SMPL group versus observation group between baseline and the 3-month follow-up (-77.8 ± 72.3 μm vs. -45.0 ± 46.9 μm, *P* = 0.049). The alteration in AMT did not differ between the two groups in either 1-month (-16.5 ± 20.1 μm vs. -19.7 ± 16.3 μm, *P* = 0.547) or 3-month (-36.9 ± 26.9 μm vs. -34.0 ± 20.1 μm, *P* = 0.678) follow-up.

**Conclusions:**

SMPL therapy led to a significant decrease in CST at the 3-month follow-up while did not significantly improve the visual acuity in patients with postoperative macular thickening following iERM surgery.

**Trial registration:**

The study was registered on Aug 27, 2020 (Trial Registration Number: ChiCTR 2000037227).

## Background

Idiopathic epiretinal membrane (iERM) represents a common ocular disorder among elderly individuals causing visual impairment and metamorphopsia [1]. iERM is characterized by the excessive growth of fibrocellular tissue that proliferates on the inner surface of the macular region, often culminating in macular pucker and edema. Modern pars plana vitrectomy (PPV) combined with membrane peeling has been considered a safe and effective technique for the treatment of iERM [2, 3]. However, in some cases, macular thickening may persist after iERM peeling and contributes to suboptimal improvement in visual acuity and metamorphopsia [4, 5]. Unlike diabetic or other vasogenic macular edema, iERM-induced macular thickening often exhibits mild inflammation component and little microvascular leakage, and thus may be refractory to conventional anti-inflammatory and anti-neovascularization treatment [6–8]. Prolonged persistence of macular thickening may cause further degeneration and functional impairment of the macula [8]. Currently, there is a lack of reliable method to achieve further structural and functional improvement in eyes subjected to ERM peeling.

In the past decade, subthreshold micropulse laser (SMPL) has gained much popularity as a safe and effective treatment alternative for patients with macular disorders [9–11]. Different from the traditional laser photocoagulation that achieve its therapeutic effects through the destruction of photoreceptors, SMPL delivers laser energy in short, intermittent, and repetitive bursts, characterized by a sublethal cellular thermal effect, which allow it to selectively act on retinal pigment epithelium (RPE) cells while averting laser-induced retinal damage [12, 13]. The duty cycle, defined as the percentage of time during which the laser is “on” within each micropulse period, allows the dissipation of accumulated heat to the retina tissue [12, 14]. Increasing evidence has demonstrated the clinical safety and effectiveness of SMPL to improve macular function in a variety of diseases, including diabetic macular edema (DME) [11, 14–19], macular edema secondary to retinal vein occlusion (RVO) [20], central serous chorioretinopathy (CSC) [21, 22], age-related macular degeneration (AMD) and inherited retinal degeneration [23–25], and open-angle glaucoma [26].

In this study, we investigate the short-term effects of SMPL on early postoperative macular thickening following surgical removal of iERM, with special attention to the changes in macular thickness and visual acuity.

## Materials and methods

### Subjects

This prospective study adhered to CONSORT guidelines and conformed to the guidelines of the Declaration of Helsinki. The research was conducted in compliance with a suitable accredited institutional review board from the Ethics Committee of Joint Shantou International Eye Center (JSIEC) of Shantou University and the Chinese University of Hong Kong, and has been registered on the Chinese Clinical Trial Registry (ChiCTR 2000037227) before the first participant is enrolled. Informed consent was obtained from every patient after an explanation of the nature and possible consequences of the study. Consecutive patients from Oct 2020 to Dec 2021 who met the following criteria were included: 1) male or female aged 40 years or above; 2) diagnosed with idiopathic epiretinal membrane and underwent combined phacoemulsification and 23-guage PPV; 3) presented with persistent macular thickening following iERM removal, which was defined as a central subfield thickness (CST) of over 250 μm based on optical coherence tomography (OCT) morphology at 1 month after the surgery; 4) only one eye of each patient was included in the study. Exclusion criteria include: 1) had a history of PPV surgery before iERM was determined; 2) combined with other ocular diseases, including high myopia (< -6.0 diopters); full/lamellar macular hole, diabetic retinopathy, retinal vascular diseases, macular degeneration, and others; 3) confound systemic diseases including uncontrolled hypertension or diabetes; 4) loss to follow-up. All participants were Asian (Chinese Han population).

### Study design

**Fig. 1:**
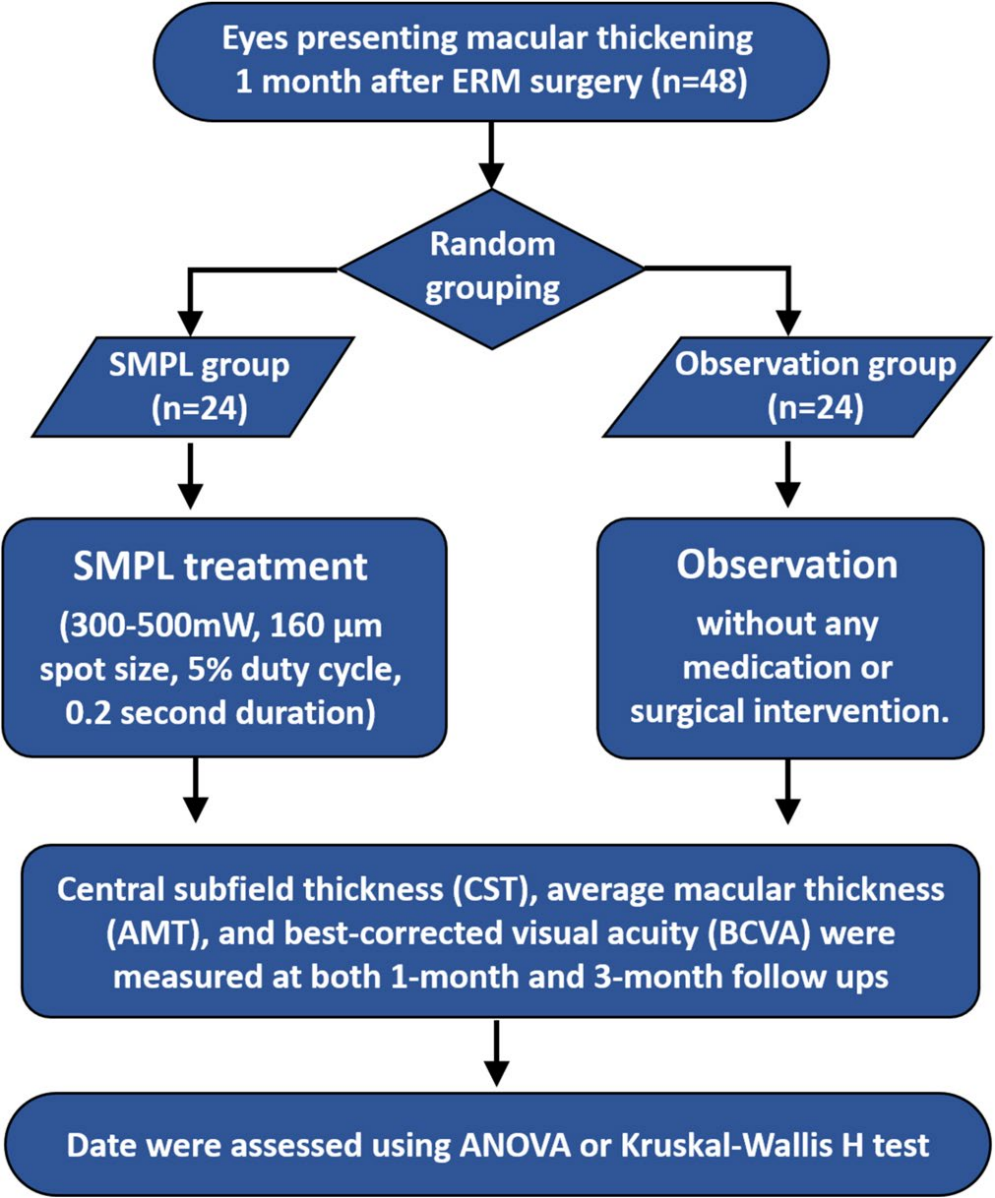
Flowchart of the study. ERM indicates epiretinal membrane; SMPL, subthreshold micropulse laser; ANOVA, analysis of variance

This was a single-center, prospective and interventional cohort study. Consecutive patients were randomly divided into two subgroups using random numbers through a Microsoft Excel spreadsheet. Patients with an odd number were assigned to the SMPL treatment group while those with an even number were arranged to the observation group. Patients in the SMPL group were offered a single subthreshold micropulse laser treatment within a few days after grouping in the absence of other treatments. Observation-only was chosen for the other group as a control (Fig. 1).

### Surgical procedures and postoperative medication

All surgical procedures were performed by the same surgeon (W. Chen). Specifically, Standard PPV was performed with vitrectomy system (Alcon Constellation Vision System; Alcon) Indocyanine green (2 mg/ml) was used to visualize the ERM and internal limiting membrane (ILM). Membrane peeling was performed using ILM forceps (Grieshaber, Alcon). The size of ILM removal was approximately 3 disk diameters. No foveal massage was conducted after peeling of ILM. Fluid/air exchange with complete air filling was performed. Triamcinolone acetonide (2 mg in 0.05 ml) was injected into the vitreous cavity at the end of the surgery. The post-operative medication was the same for both groups, which included: 0.3% levofloxacin hydrochloride eye drops, 4 times a day for one month. 1% prednisolone acetate eye drops: 4 times a day during the first week after surgery, three times a day during the second week, twice a day during the third week, and once a day during the fourth week. Tobramycin dexamethasone eye ointment, once nightly for 10 days. No topical medication was used after the SMPL treatment.

### Subthreshold micropulse laser (SMPL) treatment

SMPL was performed by the same technician. After topical anesthetic was applied, a Mainster Macular corneal contact lens (magnification factor 1.05×, Ocular Instruments, Mentor, Ohio) was placed over the cornea. SMPL (577 nm pure yellow laser, Supra Scan; Quantel Medical, Cedex, France) was then performed in 400–800 high-density/low-intensity treatment fashion, 300-500mW power, 160-µm spot size, 5% duty cycle, 0.2-second duration, with multiple and fully confluent spots throughout the macular region encompassed by the major vascular arcades. The SMPL treatment was conducted uniformly and consistently, adhering to identical parameters in all treated eyes.

### Main outcome measurement

The main outcome measures included the changes in macular thickness and best-corrected visual acuity (BCVA). All eyes were assessed using Cirrus HD-OCT (Carl Zeiss Meditec Inc, Dublin, CA, USA). Macular thickness was evaluated through the acquisition of 512 × 128 line scans. Measurement of central subfield thickness (CST), defined as the average retinal thickness within the 1-mm central scanned area, and average macular thickness (AMT), representing the average retinal thickness across the nine macular sectors within a 6-mm diameter circle centered on the fovea, was automatically obtained by the Spectral Domain (SD)-OCT system. The changes in CST/AMT were defined as the subtraction of follow-up CST/AMT values from baseline CST/AMT values. SD-OCT was performed at the time of enrollment, and at the 1-month and 3-month follow-ups. Follow-up CST and AMT measurements were taken at the same point as the baseline measurement. BCVA with Snellen chart was evaluated preoperatively and postoperatively.

### Sample size

The sample size was calculated based on significance test of difference using the formula for comparison the means of two samples. The power (1-β) was set as 0.90. α = 0.05. The CST was chosen as the main outcome measurement. According to previous literatures [27], the population standard deviation (σ) of CST was determined as 60 μm, and the minimal but significant difference (δ) was set as 80 μm. The sample size was calculated from the following equation:

$$N\;=\;\left(\frac{\left(Z_{^{\alpha}/_{2}}+\;Z_\beta\right)\cdot\sigma}\delta\right)^2\;\cdot\;\left(Q_1^{-1}\;+Q_2^{-1}\;\right)\;=\;\left(\frac{\left(1.645\;+\;1.282\right)\cdot60}{80}\right)^2\;\cdot\;\left(0.5^{-1}\;+\;0.5^{-1}\right)\;\approx22$$

In this study, 24 patients in each group would be enough to get a statistically significant result.

### Statistical analysis

Clinical data were summarized and presented as mean values and standard deviations (SD), range for continuous variables, and percentages for categorical variables. Baseline to follow-up comparisons were assessed using one-way analysis of variance (ANOVA) or Kruskal-Wallis H test (when small sample sizes or nonnormal data were present). The difference in BCVA, CST, and AMT between the two subgroups were analyzed using student-t test or Mann-Whitney U test. Chi square test was used to compare the incidence rates. BCVA were converted to logMAR units for statistical analysis. All analyses were performed using SPSS statistical software (version 25.0, SPSS Inc, Chicago, IL). *P* values of less than 0.05 were considered statistically significant.

## Results

A total of 48 eyes from 48 patients with persistent macular edema after PPV and iERM peeling were included. The patients included 16 males and 32 females, aged 63.1 ± 7.7 years old. The average LogMAR BCVA in all included eyes were 0.30 (0.28 ~ 0.52). The mean CST was 421.4 ± 80.7 μm and AMT was 317.5 ± 33.7 μm at baseline. No significant difference in baseline characteristics between the SMPL and observation groups was found (*P* > 0.05). Baseline characteristics data are shown in Table 1.

**Table 1:** Baseline demographic characteristics of subjects with persistent macular thickening following surgical removal of epiretinal membrane

	Total (*n* = 48)	SMPL (*n* = 24)	Observation (*n* = 24)	* P* value (SMPL vs. Observation)
Age/years	63.1 ± 7.7 (43 ~ 79)	64.5 ± 8.1 (43 ~ 79)	61.6 ± 7.2 (48 ~ 76)	0.198 ^t^
Male/%	33.3%	33.3%	33.3%	1.000 ^χ^
LogMAR BCVA	0.30 (0.28 ~ 0.52)	0.30 (0.22 ~ 0.54)	0.30 (0.30 ~ 0.52)	0.585 ^u^
IOP/mmHg	13.4 ± 2.7 (7.0 ~ 19.0)	13.7 ± 3.1 (8.0 ~ 19.0)	13.1 ± 2.3 (7.0 ~ 17.0)	0.496 ^t^
CST/um	421.4 ± 80.7 (266.0 ~ 569.0)	457.0 ± 70.1 (342.0 ~ 569.0)	430.5 ± 65.8 (266.0 ~ 540.0)	0.184 ^t^
AMT/um	317.5 ± 33.7 (280.0 ~ 425.0)	340.9 ± 39.0 (281.0 ~ 425.0)	330.2 ± 34.0 (280.0 ~ 405.0)	0.317 ^t^

Data were presented as median (interquartile range) for BCVA and as mean ± SD (range) for IOP, CST, and AMT

*SMPL *Subthreshold micropulse laser, *BCVA *Best-corrected visual acuity, *IOP *Intraocular pressure, *CST *Central subfield thickness, *AMT* Average macular thickness

*P*^t^, student^’^s T-test; *P*^χ^, chi square test; *P*^u^, Mann-Whitney U test

### Best corrected visual acuity (BCVA)

**Fig. 2:**
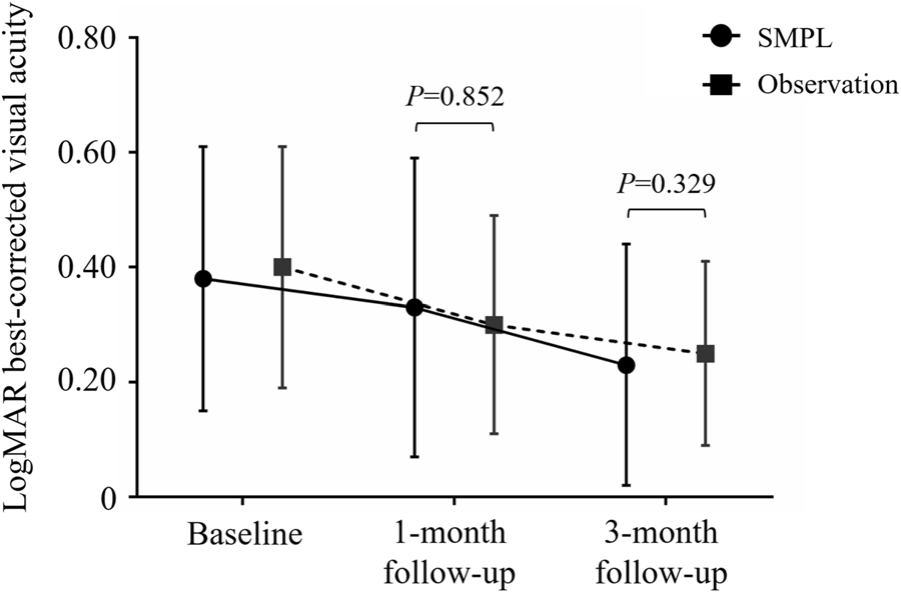
Mean best corrected visual acuity values of patients in the subthreshold micropulse laser (SMPL) group and observation group along the follow-up period. The changes were comparable (*P* > 0.05) in both groups at different time points. Data were presented as median and interquartile ranges

The logMAR BCVA showed a trend toward an improvement in both SMPL and observation groups, and was significantly better in the 3-month follow-up as compared with baseline (0.15 [0.10, 0.30] vs. 0.30 [0.22, 0.54], *P* = 0.036 for SMPL group, 0.23 [0.15, 0.30] vs. 0.30 [0.30, 0.52], *P* = 0.016 for observation group) (Table 2). No significant difference in BCVA between SMPL and observation groups was noted, either in the 1-month (0.26 [0.15, 0.52] vs. 0.26 [0.15, 0.39], *P* = 0.852) or the 3-month follow-up (0.15 [0.10, 0.30] vs. 0.23 [0.15, 0.30], *P* = 0.329) (Fig. 2). We further divided the patients into two baseline visual acuity levels (logMAR BCVA ≥ 0.4, logMAR BCVA < 0.4) and compared the changes of BCVA in subgroups. In those with a baseline logMAR BCVA of greater or equal to 0.4, there was 90% (9/10) of eyes in the SMP group while 100% (10/10) in the observation group that presented improved BCVA. In those with a logMAR BCVA of less than 0.4, the rate of visual improvement in the follow-up was 78.6% (11/14) in the SMP group and 61.5% (8/13) in the observation group. No significant difference was observed between the two subgroups.

**Table 2:** Best-corrected visual acuity in subgroups at baseline and follow-ups

	Baseline	1-month follow-up	3-month follow-up	* P* value (Baseline vs. 1-month)	* P* value (Baseline vs. 3-month)
SMPL	0.30 (0.22 ~ 0.54)	0.26 (0.15 ~ 0.52)	0.15 (0.10 ~ 0.30)	0.338 ^h^	0.036 ^h^
Observation	0.30 (0.30 ~ 0.52)	0.26 (0.15 ~ 0.39)	0.23 (0.15 ~ 0.30)	0.170 ^h^	0.016 ^h^

Data were presented as median (interquartile range)

*SMPL* Subthreshold micropulse laser

*P*^h^, Kruskal-Wallis H test

### Macular thickness

**Fig. 3:**
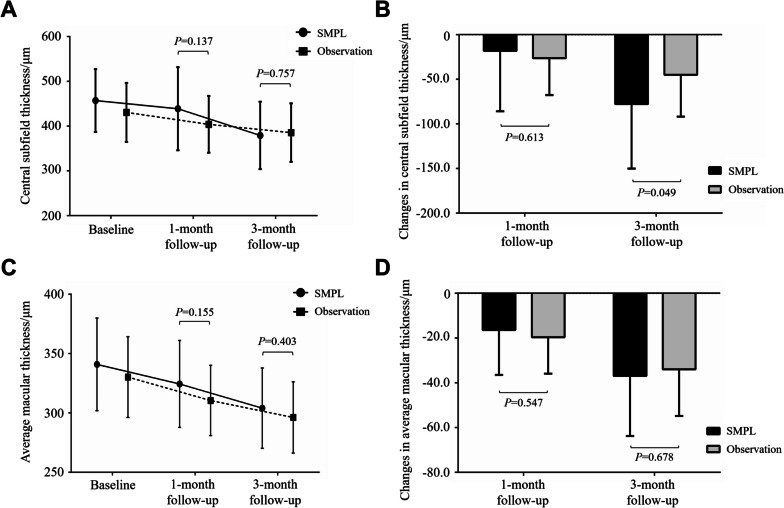
Mean central subfield thickness (CST) and average macular thickness (AMT) values in the micropulse laser (SMPL) group and observation group along the follow-up period. **A** The CST values between the two groups were comparable (*P* > 0.05). **B** SMPL group showed significant changes in CST between follow-up and baseline in the 3-month follow-up (*P* = 0.049) compared with observation group. **C**-**D** Both the AMT values (**C**) and the changes in AMT between follow-up and baseline (**D**) were comparable (*P* > 0.05) in both groups

The central subfield thickness (CST) showed a significant reduction from baseline to the 3-month follow-up in both groups (379.2 ± 75.3 μm vs. 457.0 ± 70.1 μm, *P* = 0.004 for SMPL group; 385.5 ± 65.3 μm vs. 430.5 ± 65.8 μm, *P* = 0.049 for observation group) (Table 3). No significant difference in CST values was found between the SMPL and observation groups at the 1-month (438.8 ± 92.9 μm vs. 404.0 ± 63.5 μm, *P* = 0.137) or the 3-month follow-up (379.2 ± 75.3 μm vs. 385.5 ± 65.3 μm, *P* = 0.757) (Fig. 3A). We further analyzed the changes in CST between the two subgroups. In the 3-month follow-up, a greater reduction in CST was noted in the SMPL group than observation (-77.8 ± 72.3 μm vs. -45.0 ± 46.9 μm, *P* = 0.049) (Fig. 3B). In addition, there was a higher proportion of patients in the SMPL group who had decreased CST from baseline to the 3-month follow-up, as compared with the observation group (23/24, 95.8% vs. 18/24, 79.2%, *P* = 0.026).

**Table 3:** Changes in macular thickness in subgroups at baseline and follow-ups

	Group	Baseline	1-month follow-up	3-month follow-up	* P* value (Baseline vs. 1-month)	* P* value (Baseline vs. 3-month)
CST	SMPL	457.0 ± 70.1 (342 ~ 569)	438.8 ± 92.9 (273 ~ 635)	379.2 ± 75.3 (194 ~ 482)	0.711 ^a^	0.004 ^a^
	Observation	430.5 ± 65.8 (266 ~ 540)	404.0 ± 63.5 (309 ~ 543)	385.5 ± 65.3 (274 ~ 555)	0.339 ^a^	0.049 ^a^
AMT	SMPL	340.9 ± 39.0 (281 ~ 425)	324.4 ± 36.6 (265 ~ 398)	304.0 ± 33.8 (253 ~ 374)	0.269 ^a^	0.002 ^a^
	Observation	330.2 ± 34.0 (280 ~ 405)	310.5 ± 29.6 (276 ~ 390)	296.2 ± 30.0 (267 ~ 386)	0.082 ^a^	0.001 ^a^

Data were presented as mean ± SD (range)

*CST *Central subfield thickness, *AMT *Average macular thickness, *SMPL *Subthreshold micropulse laser, *SD *Standard deviation

*P*^a^, One-Way ANOVA test

**Fig. 4:**
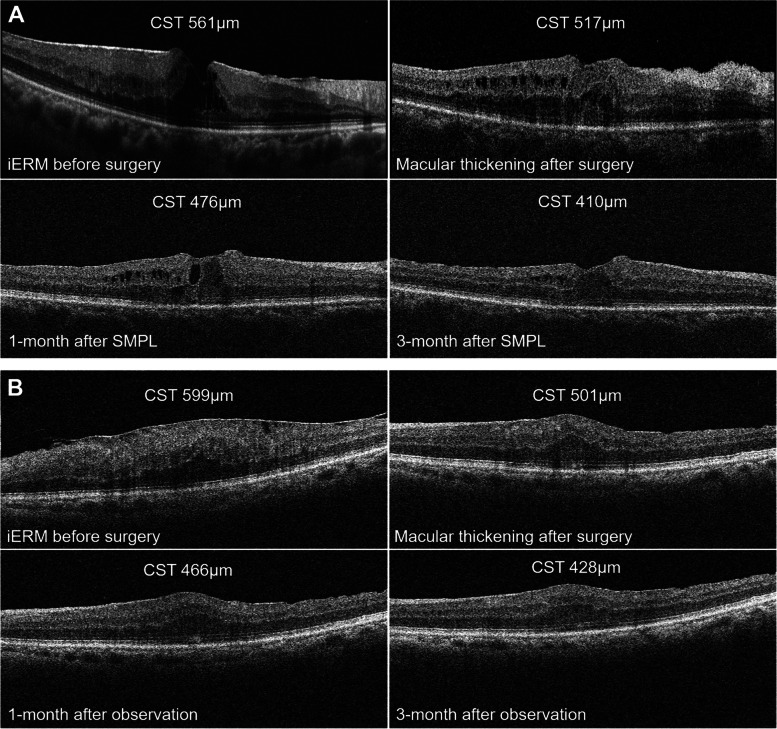
Representative OCT morphology of patients in the subthreshold micropulse laser (SMPL) group and observation group along the follow-up period. **A** SMPL group; **B** Observation group

The average macular thickness (AMT) was consistent with the CST results comparing the two groups, which showed a trend toward an improvement during the follow-up period, with a significant reduction in the 3-month follow-up from baseline (304.0 ± 33.8 μm vs. 340.9 ± 39.0 μm, *P* = 0.002 for SMPL group; 296.2 ± 30.0 μm vs. 330.2 ± 34.0 μm, *P* = 0.001 for observation group) (Table 3). However, no significant difference in AMT between the SMPL and the observation group was observed in the 1-month (324.4 ± 36.6 μm vs. 310.5 ± 29.6 μm, *P* = 0.155) or the 3-month follow-up (304.0 ± 33.8 μm vs. 296.2 ± 30.0 μm, *P* = 0.403) (Fig. 3C). There was also no difference in the reduction of AMT from baseline to the follow-ups between the two subgroups either in the 1-month (-16.5 ± 20.1 μm vs. -19.7 ± 16.3 μm, *P* = 0.547) or 3-month (-36.9 ± 26.9 μm vs. -34.0 ± 20.1 μm, *P* = 0.678) follow-ups compared to baseline (Fig. 3D). Representative OCT findings of patients in the SMPL and observation groups were shown in Fig. 4.

### Safety

No eyes in this study developed recurrent ERM, full/ lamellar macular hole, or other macular complications requiring additional surgery during the 3-month follow-up period. There was no visible side effect of SMPL to the neuroretinal or RPE in any eye during the follow-up period, such as laser scaring, outer retinal changes or other significant changes in retinal morphology, as evaluated by clinical examination, fundus autofluorescence photography, and SD-OCT.

## Discussion

iERM is determined as idiopathic with the absence of other ocular diseases [28]. Several factors, including activation and migration of glial cells, fibroblasts, hyalocytes, as well as the involvement of cytokines and growth factors, have been elucidated as contributing elements in the formation of contractile membranes [1]. PPV following membrane peeling has been considered a safe and effective treatment strategy to improve visual function and metamorphopsia in patients with iERM [3, 29]. However, a notable subset of patients may have macular thickening persisted with limited visual improvement after surgery [30]. Although spontaneous improvement of the macular thickening may occur over the course of several months following surgical membrane peeling, a subset of patients still suffers from persistent macular thickening and limited visual improvement. The pathomechanism responsible for postoperative macular thickening remains unclear, with hypotheses suggesting residual effect of tangential epiretinal traction or microvascular leakage [4, 31]. However, fundus fluorescein angiography demonstrated little to no leakage in these eyes, which did not support the latter hypothesis [27]. Given the absence of angiographic leakage in this process, medical interventions, such as corticosteroids or anti-vascular endothelial growth factor (VEGF), are often ineffective [32, 33]. We also contemplate whether the postoperative macular thickening in these patients would be indicative of Irvine-Gass syndrome. However, the incidence rate of Irvine-Gass syndrome is relatively low, ranging from 0.1 to 2.35% [34]. In addition, Irvine-Gass syndrome is hypothesized to result from vitreoretinal traction on the macula following cataract surgery, leading to alteration in macular capillary permeability and subsequent cystoid macular edema [34]. In this study, all eyes underwent vitrectomy with complete induction of posterior vitreous detachment. Therefore, the possibility of Irvine-Gass syndrome involvement in this cohort is relatively low, although it cannot be completely ruled out.

The potential of SMPL as a treatment strategy for macular diseases is mainly based on its ability to orchestrate and reactive RPE cells [12, 13]. Using a “low-intensity/high-density” paradigm, SMPL induces activation of heat shock proteins in the RPE, which triggers a physiologic “repair” or “reset” to normalize RPE cell function and cytokine/chemokine expression [13, 35–37]. Recent studies also demonstrated the roles of SMPL in other target cells, including the inhibition of microglial activation and neuroinflammation, and down-regulation of Müller cell-derived VEGF expression [36, 38]. Besides, SMPL played anti-apoptotic and neuroprotective effects on retinal Müller cell and ganglion cells, and was found to increased tissue nitrous oxide and improves mitochondrial function [39, 40]. These findings open up new ideas for the potential of SMPL in improving retinal metabolism, morphology and function.

In this study, we introduced SMPL as an adjunctive therapy for patient with early postoperative macular thickening subsequent to iERM peeling. It was noted that both SMPL-treated and observation groups exhibited visual improvement during the 3-month follow-up, whereas SMPL treatment failed to gain additional visual benefit. This observation was consistent with several previous studies regarding to diabetic macular edema in which SMPL has shown visual acuity outcomes comparable to either conventional laser treatments or non-treated controls [41–43]. Although it does not bring additional visual acuity benefits, SMPL can improve macular microstructure by resetting RPE function and regulating glial cell activation, resulting in enhanced contrast sensitivity [42, 44], which is also an aspect of visual function.

Concerning macular thickness, we observed a decrease in CST, slight but significant, in the 3-month follow-up in eyes treated with SMPL than non-treated controls. This finding is consistent with most previous literatures [45–55], which supports the notion that SMPL induces enhancements in macular architecture. In particular, the decrease in CST elicited by SMPL was greater at the 3-month follow-up than the 1-month follow-up, indicating a delayed treatment effect of SMPL in eyes subjected to iERM peeling. The decoupling of functional and anatomical changes in the SMPL treatment process has been previously documented in eyes with DME, CSC, and AMD [45, 55–58]. In this study, it was likely due to a relatively short follow-up period within which the improvement in visual acuity had not yet been uncovered. Another possibility was that we used a single SMPL treatment. We are left to ponder whether a repeated SMPL treatment might help to amplify or maintain both structural and functional improvements.

It was also noted that the CST demonstrated an improvement following SMPL while the AMT did not, which was inconsistent with a previous study showing significant changes in average retinal thickness instead of CST after SMPL treatment [27]. The reason was unclear. In this study, a ceiling effect may account for this finding, as the average baseline CST in this study (421.4 μm) markedly exceeded the baseline AMT (317.5 μm), leaving more space for CST improvement.

The current study was limited by its single-center design, relatively small sample size, a single SMPL treatment, and short follow-up period. The latter one was associated with poor long-term patient compliance during the long-term visit, possibly due to a stable disease condition as well as the COVID-19 restrictions. As some studies showed the SMPL effects in DME begin to be significant at 3 months post treatment, extending the follow-up time may reveal potential differences between the groups in this study. In addition, there was lack of functional evaluation other than visual acuity, such as microperimetry, mf-ERG and contrast sensitivity, since patients could detect improvement in different functional tests after SMPL treatment. Further investigation with a longer follow-up period and more comprehensive visual function assessment is warranted.

## Conclusions

While SMPL has demonstrated efficacy in treating various macular diseases, research on its potential restorative role in early postoperative macular thickening following iERM peeling is lacking. In this prospective study, we observed a significant decrease in CST in ERM-peeled eyes treated with SMPL, although there was no improvement in short-term BCVA compared to the observation group. Despite the less satisfactory visual outcome, SMPL may serve as an adjuvant treatment for early postoperative macular thickening following ERM peeling. It offers hope for improving RPE function and metabolism, particularly in patients who do not respond adequately to anti-VEGF or anti-inflammatory therapy.

## Data Availability

All data supporting these findings are contained within this manuscript.
